# Is Long-term Exposure to Air Pollution Associated with Episodic Memory? A Longitudinal Study from Northern Sweden

**DOI:** 10.1038/s41598-017-13048-1

**Published:** 2017-10-06

**Authors:** Anna Oudin, Bertil Forsberg, Nina Lind, Steven Nordin, Daniel Oudin Åström, Anna Sundström, Maria Nordin

**Affiliations:** 1 Occupational and Environmental Medicine, Public Health and Clinical Medicine, Umeå University, 901 87 Umeå, Sweden; 2 Department of Psychology, Umeå University, 901 87 Umeå, Sweden; 3 Family medicine, cardiovascular epidemiology and lifestyle, Lund University, 205 02 Malmö, Sweden; 4 Centre of Demographic and Ageing Research (CEDAR), Umeå University, Umeå, Sweden

## Abstract

Associations between long-term exposure to ambient air pollution and cognitive function have been observed in a few longitudinal studies. Our aim was to investigate the association between long-term exposure to air pollution and episodic memory, a marker of early cognitive decline. We used data from the Betula study in Northern Sweden, and included participants 60 to 85 of age at inclusion, 1,469 persons in total. The participants were followed for up to 22 years, five years apart between 1988 and 2010. A composite of five tasks was used as a measure of episodic memory measure (EMM), and the five-year change in EMM score (ΔEMM) was calculated such that a participant could contribute with up to four measurement pairs. A Land Use Regression Model was used to estimate cumulative annual mean of NOx at the residential address of the participants (a marker for long-term exposure to traffic-related air pollution). There did not seem to be any association between exposure to traffic air pollution and episodic memory change, with a ΔEMM estimate of per 1 µg/m3 increase in NOx of 0.01 (95% Confidence Interval: −0.02,0.03). This is in contrast to a growing body of evidence suggesting associations between air pollution and cognitive function.

## Introduction

Air pollution is a well-known cause of mortality and morbidity worldwide^1^, and as stated in a recent review, evidence is building up for air pollution to have a direct causal effect on cognition^2^. The mechanisms are not entirely known, but particulate air pollution may pass through the blood-brain-barrier, pass through the olfactory bulb, cause systemic inflammation and activate gliacells^3–6^, and air pollutants have been linked to brainstem auditory nuclei pathology and delayed brainstem auditory evoked potentials^7^ and to olfactory dysfunction and olfactory bulb pathology^8^. Cross-sectional associations between air pollution concentrations and various markers of cognitive function have been observed in many studies^9–15^. In the Nurses’ Health Study Cognitive Cohort however, which included 19,409 elderly women in the US, long-term exposure to particles preceding baseline cognitive testing was assessed longitudinally^16^. The main outcome measure was cognition via validated telephone assessments at approximately 2-year intervals. Long-term exposure was found to be associated with faster cognitive decline, and a 10 μg/m^3^ increase in long-term particulate matter exposure was found to be the cognitive equivalent to aging by approximately 2 years. The associations with exposure to fine and coarse particles were examined separately and found to be of similar magnitude. In another study, air pollution (PM_2.5_) was associated with smaller total cerebral brain volume which is a marker of age-associated brain atrophy, and with higher odds of covert brain infarcts in dementia- and stroke-free persons^17^. Also, exposure to fine particulate matter may contribute to white matter loss in older women^18^. However, in a study in a study area where levels of fine particulate matter were below the EPA standard, there did not seem to be any association between air pollution and small vessel disease or neurodegeneration^19^. Associations between air pollution and incident dementia have been reported by us^20^ and others^21–23^. In the US, the number of people affected by late-onset neurodegenerative disorders such as Alzheimer’s disease is predicted to triple within 40 years unless preventive measures are developed^24^. It is therefore important to identify risk factors for such disorders. Episodic memory is typically regarded as highly age-sensitive, and cognitive decline is often first observed in episodic memory^25^. Episodic memory is, therefore, a marker of interest in capturing early stages of cognitive decline.

Earlier studies on air pollution and cognitive function have been cross-sectional with a few exceptions, with obvious limitations in inferring causality. Furthermore, previous studies have mainly been conducted in areas where air pollution levels are rather high. The aim of the present study was to investigate possible associations between chronic exposure to air pollution and longitudinal changes in episodic memory in an area with rather low levels of air pollution.

## Results

There were 1,469 persons 60 years or older who had participated in the Betula study at least twice during the study (Table 1), and they contributed with a total of 2,516 measurement pairs. The individuals were followed for a mean of 8.6 years (standard deviation (SD): 4.4 years). The mean level of Nitrogen Oxides (NOx) during follow-up was 20.9 μgm^−3^ (SD: 16.1 μgm^−3^), and the mean baseline Episodic Memory Measure (EMM) (EMM at the first in a pair of two consecutive tests, ${EMM}_{T}$) was 33 (SD: 9.8). The crude mean decrease in the EMM score per year of age was −0.47 (95% confidence interval (CI): −0.52, −0.42), which changed to −0.52 (95% CI: −0.57, −0.46) when the number of tests a study subject had taken, and test occasion was added to the model as independent variables. As anticipated, *ΔEMM* was highly dependent on age and on the number of participations in the Betula study (Table 1).

**Table 1:** Background variables and episodic memory composite differences (Δ*EMM*) calculated as the difference between two consecutive tests (a negative value denotes a decline over time) for 2516 measurement pairs. “Number of tests per person” denotes the total number of tests a person undertook during follow-up. “Age” denotes the age at the first test in a pair, and the same is true for all other variables.

	N (%)	Mean (Δ*EMM*)	Mean NOx (µgm^−3^)
Age			
60	609 (24)	−0.76	19.5
65	600 (24)	−1.71	20.3
70	523 (21)	−3.25	21.1
75	425 (17)	−4.21	22.9
80	268 (11)	−5.84	25.9
85–95	91 (4)	−5.44	23.2
Number of tests per person			
2	632 (25)	−3.38	21.6
3	476 (19)	−3.82	21.2
4	831 (33)	−2.37	20.4
5	577 (23)	−1.93	23.1
Sex			
Male	1,429 (57)	−2.93	22.1
Female	1,087 (45)	−2.62	20.5
Education			
Missing	122 (5)	−3.29	26.0
Low	1,483 (60)	−2.90	20.9
Medium	484 (19)	−2.61	20.9
High	427 (17)	−2.52	22.3
Smoking			
Missing	3 (0)	−6.0	14.3
No	1,379 (55)	−2.90	21.0
Yes or former	1,134 (45)	−2.69	21.9
Physical Activity			
Missing	22 (1)	−4.50	21.3
No	581 (23)	−3.39	21.9
Yes	1,913 (76)	−2.59	21.3
Living with someone			
Missing	202 (8)	−4.28	25.6
No	713 (28)	−3.63	24.6
Yes	1,601 (64)	−2.24	19.5
Work status			
Missing	193 (8)	−2.50	22.4
No	1,762 (70)	−3.35	22.0
Yes	561 (22)	−1.15	19.3

In the crude model, there was a small association between *NOx* and *ΔEMM*, with an estimate of −0.18 (95% CI: −0.32, −0.004) per 1 µg/m^3^ increase in NO_x_. However, the association was no longer present in Model 1 or Model 2, with an estimate in Model 2 (where variables for age, test occasion, number of total tests, education, sex, smoking, BMI, physical activity, cohabitation, and work status was included in the model as independent variables) of 0.005 (95% CI: −0.002, 0.027; Table 2). Age was the single variable that explained most of the association observed in the crude model, and this is not surprising given a strong association between age and air pollution (data not shown).

**Table 2:** Change in episodic memory composite differences between two consecutive tests (Δ*EMM*) for 1,469 persons in association with air pollution concentrations (NO_x_) at the home address during follow-up analyzed with Generalized Estimation Equations with repeated measurement per individual. Results are presented as Δ*EMM* with 95% confidence intervals (95% CIs), n is the number of measurement pairs. A negative Δ*EMM* denotes a decrease over time.

NO_x_	Crude model, n = 2,516	Model 1, n = 2,516	Model 2, n = 2,059
	Δ*EMM* (95% CI)	Δ*EMM* (95% CI)	Δ*EMM* (95% CI)
Quartile 1^a^	ref	ref	ref
Quartile 2	−0.43 (−1.11,0.24)	−1.22 (−2.94,0.49)	−1.33 (−3.15,0.48)
Quartile 3	−0.64 (−1.32,0.05)	−0.78 (−2.53,0.97)	−0.72 (−2.55,1.10)
Quartile 4	−0.91 (−1.54, −0.27)	−1.69 (−3.36,−0.022)	−1.45 (−3.22,0.31)
Linear^b^	−0.18 (−0.32, −0.004)	0.001 (−0.020, 0.02)	0.005 (−0.018,0.027)

^a^The quartile limits were 8.4 µgm^−3^, 15.4 µgm^−3^, and 24.0 µgm^−3^. ^b^Estimate per 1 µgm^−3^ increase in NO_x_ Model 1 includes variables for NO_x_, age, test occasion, number of total tests, and a cross-product between NO_x_ and test occasion Model 2 is the same as Model 1, but also included variables for education, sex, smoking, BMI, physical activity, living with someone, and work status.

The interaction term between *T* and *ntest* only influenced the estimates marginally (data not shown), and including baseline EMM into the models did not have any substantial influence on the estimates of the present study (data not shown). Baseline air pollution was not associated with baseline EMM (data not shown), and baseline EMM score did not modify the (lack of) association between EMM change and air pollution. The change from baseline analysis did not indicate any associations between EMM change and baseline air pollution concentrations. By including only baseline concentrations or only the concentration at the first measurement pair, instead of the cumulative air pollution measure did not alter the results. There is a clear gradient in EMM change by age (Supplementary Table 1). EMM change by the interaction-term between T and NOx can be seen in Supplementary Table 2.

## Discussion

We observed no overall association between traffic air pollution concentrations at home at baseline and cognitive decline measured as episodic memory score. Our results are somewhat in contrast with a previous study where a 10 μg/m^3^ increment in long-term particulate matter exposure was found to be cognitively equivalent to aging by approximately 2 years^16^. There are several potential explanations for the differences in findings; our study sample was smaller, we modeled exposure to traffic air pollution using NOx, not particulate matter as a marker of air pollution with other sources than vehicle exhaust, and we used a composite score of episodic memory as outcome whereas the other study used another measure of cognitive decline. Also, our study was conducted in an area with rather low levels of air pollution, and we cannot rule out that associations between air pollution and episodic memory decline are present at higher levels of air pollution.

Interestingly, our results are also in contrast with an increasing number of cross-sectional studies have shown associations between ambient air pollution at home and cognitive status in adults^9,10,12–15,21,22,26^. In only one study (to our knowledge) was there no clear association between cognition and air pollution^10^, which is similar to what we observed in the present study, because not only were there no longitudinal associations, there were no cross-sectional analysis associations between baseline NO_x_ and episodic memory. It is important to note that cross-sectional analyses have severe limitations when assessing causality, and studies on cognitive outcomes are probably especially sensitive. Residential choices might, for example, be highly dependent on cognitive status. It is, therefore, difficult to conclude whether associations observed between cognitive outcomes and air pollution concentrations are due to a causal effect or due to inherent study bias.

We chose to study a composite episodic memory score as a measure of cognitive status. Other studies on air pollution and cognition have focused on a range of different measures. It is impossible to rule out that another choice of outcome variable would have been associated with air pollution, but because our hypothesis was derived from a desire to understand the association between dementia incidence and air pollution in our study area^20^, and because episodic memory decline is a hallmark manifestation of dementia^27,28^, we considered episodic memory score to be the most relevant measure of cognition in our study setting.

There are both strengths and weaknesses of the present study that should be discussed. The exposure assessment model (of NOx) had a high resolution and validity and has been used in a large number of previous studies, for example, the ESCAPE studies^29–32^. A potential concern is that we only used NOx as a marker of exposure to air pollution. It would have been desirable to study for example PM_2.5_ also, which we did not have access to. However, NO_x_ is a well-known marker of vehicle exhaust, and in order to compare the results of the present study with our results on NO_x_ and incident dementia, NOx was likely the most relevant pollutant to study. Exposure misclassification must be considered in any study on long-term exposure to air pollution. We used exposure data from 2009 and assumed that differences (contrasts) in exposure would be similar back in time over the follow-up period (the first recruitments took place in 1988–1990), and this could be a source of exposure misclassification. A recent study on mice suggested an age-ceiling effect by exposure to air pollution on selective changes in the hippocampal CA1 region. If that translates to humans, we may have missed the relevant exposure window in the present study. Another potential source of exposure misclassification is that sources of air pollution other than traffic, for example, domestic wood burning, more common where there is less traffic, is not well represented in the NO_x_ exposure model. A recent study suggested traffic as the relevant source of air pollution affecting cognitive development^33^, but ambient particulate matter from domestic wood burning is a substantial contributor to the air pollution mix in our study area. The density of domestic wood burning was not available as an explanatory variable in the LUR model. Yet another potential source of exposure misclassification is the fact that ambient exposure was modeled at the home address and exposure at work or indoor exposure were not taken into account. Although these are obvious sources of exposure misclassification, we used the same approach as in many other air pollution epidemiology studies where we have been able to observe associations. For example, we observed strong associations between dementia incidence and air pollution exposure using identical exposure data within the same study sample as the present study^20^. We believe, therefore, that exposure misclassification is an unlikely explanation for the negative results of the present study, at least if any true causal effects between traffic air pollution and episodic memory decline are strong.

Another potential source of bias is that we did not take short-term exposure into account. In a previous study from London, the authors observed a decrease in mental efficiency when adults tested in London were breathing air pumped from the street compared to clean air^34^. Furthermore, human experimental exposure studies provide very limited data on the acute effects of air pollution exposure on the brain, but increased activity in the left frontal cortex during and after diesel exhaust exposure has been observed in one experimental study^35^. It would therefore have been desirable to control for short-term exposure to air pollution, for example, air pollution concentrations on the day of the testing. Unfortunately, measuring station data were not available for the entire study period and we could not control for short-term exposure to air pollution.

The present study has a major strength in its longitudinal design and the high-quality data from the Betula study. However, studies on cognitive outcomes are especially susceptible to selection bias since cognitive decline is a strong predictor of increased morbidity, mortality and attrition^36^. To cause bias in the present study, selection should be influenced by both air pollution levels and episodic memory decline. We have previously observed air pollution concentrations to be associated with mortality in the Betula study, which theoretically could have caused selection bias in the present study^29^. However, loss to follow-up for other reasons was very low^11^, we did not observe any strong cross-sectional associations between air pollution and episodic memory, and baseline episodic memory did not have any substantial influence on our effect estimates which in all may imply that selection bias as a major explanation for our null findings is not very plausible. However, there were tendencies for the number of tests in total to be related to air pollution exposure, where the persons who participated all five occasions in Betula had somewhat higher NOx values than remaining participants (Table 1). This is not due to age, since NOx tended to increase with age (Table 1). It is puzzling that we observed a strong associations between incident dementia and air pollution, but in the same cohort episodic memory decline does not seem to be associated with air pollution. In our study on dementia in association to air pollution however^20^, we saw that a large proportion of the participants with dementia only participated in the Betula study once or twice times. An explanation for the lack of findings in the present study may thus be that participants with a strong ongoing decline may not return to Betula, which means that we won’t detect their cognitive decline.

In conclusion, in one of the first longitudinal studies on air pollution and change in cognitive status, we observed no overall association between air pollution exposure at the home address and change in episodic memory over time. Our findings are in contrast to a growing body of evidence for associations between air pollution and cognitive status.

## Material and Methods

### Study area and the Betula study

The study area, Umeå municipality, had around 120,000 inhabitants in 2016 and is the largest city in northern Sweden. The air quality is generally good, for example the urban background annual mean level of PM_2.5_ is around 5 µg/m_3_. In the central parts of the city however, the EU air quality limit for nitrogen dioxide (NO_2_) is exceeded due to heavy vehicle traffic. Data on the study subjects originated from the already existing Betula study, which is described in detail elsewhere^37^. In short, Betula was first initiated to investigate health and cognition in an aging population, including early signs and potential risk factors of cognitive decline and dementia in adulthood and late life. The first data collection (T1) took place in 1988–90 when a cohort consisting of 1,000 participants in ten age cohorts (35, 40, 45, 50, 55, 60, 65, 70, 75, and 80 years) was recruited with 100 participants randomly sampled in each age cohort. On the first follow-up occasion (T2) in 1993–95, two new cohorts were added. At present, there have been four additional follow-ups and recruitments: T3 (1998–2000), T4 (2003–2005), T5 (2008–2010), and T6 (2013–2015). At T5, the first cohort had been tested five times. The participants in the Betula protocol go through a thorough health examination and an extensive interview regarding lifestyle and health together with a range of cognition tests. We excluded participants 55 years or younger at baseline.

### Episodic Memory Measure

Episodic memory is a form of declarative memory and is the memory of specific events. Episodic memory is typically regarded as highly age-sensitive, and cognitive decline is often first observed in episodic memory^38,39^. We used the episodic memory measure (EMM) previously employed in the Betula study^40^ that consists of the following five tasks: immediate free recall of 16 visually and orally presented short sentences, delayed cued recall of nouns from the previously presented sentences, immediate free recall of 16 enacted sentences, delayed cued recall of nouns from the enacted sentences, and immediate free recall of a list of 12 orally presented nouns.

### Exposure measure

All of the study subjects’ home addresses each year of follow-up were geocoded using information from the nationwide Swedish Population Registry. A Land Use Regression (LUR) model was used to estimate the annual mean levels of nitrogen oxides (NO_x_) at each address. NOx is a well-known marker of traffic-related air pollution^41,42^. We constructed our model using the same principles and geographical variables as in the large-scale European Study of Cohorts for Air Pollution Effects (ESCAPE)^31^. We used data from approximately 40 monitoring sites that represented a wide range of traffic conditions in residential, industrial, commercial, and rural locations. The final model explained 76% of the variation in the measured values. The baseline year of the LUR model was 2009.

We used the mean NOx concentration at a study subject’s home address between the measurement pairs as a marker for long-term exposure to air pollution, and we took any address changes during follow-up into account. During the years since the start of follow-up, infrastructure has changed substantially in Umeå, as well as emission factors and car usage, meaning that the quality of exposure assessment at the beginning of follow-up is questionable. One alternative way of exposure assessment is to use back-extrapolation of data by using scaling factors based on a monitoring station (to take into account changing air quality over time). We decided to use such back-extrapolation as a sensitivity analysis, similar to what has been done in ESCAPE. We collected emission data and data on daily concentrations of NO_2_ from 1990 onwards from an urban background measuring station in Umeå. In additional analyses, we stratified the data by the time point of the first visit to the Betula study (T1–T4) to control for time-dependent exposure misclassifications. In two different sensitivity analysis, we used only baseline concentrations, and the concentration at the first measurement pair only.

### Statistical analysis

The change in the EMM (*ΔEMM*) was calculated as the absolute difference between two consecutive tests as:

1 $${\rm{\Delta }}EMMi=EM{M}_{iT+1}-EM{M}_{iT}$$

where *EMMi*_*T*_ is the episodic memory measure at a given time point T (T = 1–4) for individual i, and ${{EMM}}_{{iT}+1}$ is the EMM at the time of the next participation to the Betula study.

The association between the change in episodic memory score over time and NO_x_ level at the study subjects’ homes was assumed to be linear and was in Model 1 analyzed using repeated measures with Generalized Estimating Equations taking repeated measurements within individuals into account according to Equation 2:

2 $$\Delta EMMi\, \sim \,{\beta }_{1}\cdot NOxi+{\beta }_{2}\cdot agei+{\beta }_{3}\cdot ntesti+{\beta }_{4}\cdot Ti+{\beta }_{5}\cdot {\beta }_{5}NOxi\cdot Ti$$

where *NOx*_*i*_ denotes the modeled annual mean NOx_i_ at individual *i*’s home between two consecutive tests, *age*_*i*_ denotes a categorical variable (60–85 + years with five-year intervals and 85 + denotes 85–95 years), *ntest*_*i*_ denotes the number of tests a study subject had undertaken at the end of follow-up (maximum 5, minimum 2), and *T*_*i*_ denotes the baseline time point (maximum 4, minimum 1). NOx_i_ was analyzed both as a categorical variable in quartiles and as a continuous variable. We chose an autoregressive covariance matrix for the repeated measurements per participant *i* for the main analysis.

All regression analyses were run in three separate models. In the crude model, *NOx* was the only independent variable. In Model 1, *age*_*i*_, T1–T4, ${{EMM}}_{{Ti}}$, and *ntest*_*i*_ were introduced into the model as categorical variables (Equation 2). It is important to control for cohort and retest effects^43^, therefore we also included across-product between T and ntest_i_ in Model 1. Model 2 included all variables in Model 1, and also variables for education (low, medium, high), smoking (yes/no), Body Mass Index (BMI), work status (working or not), cohabitation (living with someone or not), sex, and physical activity (rarely or at least weekly). *NO*_*xi*_ was entered both as a continuous variable and as a categorical variable, in quartiles, with quartile limits of 8.4 µgm^−3^, 15.4 µgm^−3^, and 24.0 µgm^−3^.

We chose not to include baseline episodic memory in the main analysis^36,44^, but it was added to the statistical models in a sensitivity analysis. We also removed the cross-product between T and ntest in another sensitivity analysis. We also did an analysis where we stratified the main analysis based on baseline EMM. There were too many missing observations in the data on daily variations in NO_2_ from the measuring station to be able to adjust appropriately for short-term effects, so this was omitted from the analysis. In another analysis we analyzed the association between NOx and EMM in a cross-sectional analysis of Sample 2 and 3 including age, ntest, T and NO_X_ and NO_X_*T in the models. In an extra analysis, we did a change-from-baseline analysis which was analyzed with Generalized Estimation Equations.

All participants in the Betula study gave informed consent and the study was approved by the Regional Ethical Review Board at Umeå University. The methods were carried out in accordance with the approved guidelines and regulations. All analyses were performed in the SAS v9.2 software package.

## Electronic supplementary material

Supplementary File
