# Supplementary material for: Is Long-term Exposure to Air Pollution Associated with Episodic Memory? A Longitudinal Study from Northern Sweden
Source: Sci Rep. 2017 Oct 6;7:12789. doi: 10.1038/s41598-017-13048-1 (PMC5630578; doi:10.1038/s41598-017-13048-1)
Supplement: Supplementary file 1 — Supplementary File [file 41598_2017_13048_MOESM1_ESM.pdf]

Supplementary Files to the manuscript with the title: "Is Long-term Exposure to Air Pollution Associated with Episodic Memory? A Longitudinal Study from Northern Sweden"

Authors: Anna Oudin[1*], Bertil Forsberg[1], Nina Lind[2], Steven Nordin[2], Daniel Oudin Åström[3], Anna Sundström[2], Maria Nordin,[2]

[1]Occupational and Environmental Medicine, Public Health and Clinical Medicine, Umeå University, 901 87 Umeå, Sweden

[2]Department of Psychology, Umeå University, 901 87 Umeå, Sweden

[3]Family medicine, cardiovascular epidemiology and lifestyle, Lund University, 205 02 Malmö, Sweden

Supplementary Table 1. Change in episodic memory composite differences between two consecutive tests (ΔEMM) by age for 1,469 persons in association with air pollution concentrations (NO_x) at the home address during follow-up analyzed with a Mixed Linear Model with repeated measurement per individual. Results are presented as ΔEMM with 95% confidence intervals (95% CIs), n is the number of measurement pairs. A negative ΔEMM denotes a decrease over time.

Age	Model 1, n = 2,516<br>ΔEMM (95% CI)	Model 2, n = 2,059<br>ΔEMM (95% CI)
60	Ref	Ref
65	-0.70 (-1.54-0.14)	-0.53 (-1.59-0.53)
70	-2.07 (-2.84- -1.30)	-2.11 (-3.19- -1.03)
75	-2.81 (-3.66- -1.96)	-2.57 (-3.75- -1.39)
80	-4.39 (-5.43- -3.35)	-4.66 (-6.09- -3.22)
85-95	-3.78 (-5.29—2.27)	-3.49 (-5.40- -1.58)

Model 1 includes variables for NO_x, age, test occasion, number of total tests, and a cross-product between NO_x and test occasion

Model 2 is the same as Model 1, but also included variables for education, sex, smoking, BMI, physical activity, living with someone, and work status.

Supplementary Table 2. Change in episodic memory composite differences between two consecutive tests (ΔEMM) by the interaction between time point (T=1-3, 4 is the reference) and NOx quartile for 1,469 persons in association with air pollution concentrations (NO_x) at the home address during follow-up analyzed with a Mixed Linear Model with repeated measurement per individual. Results are presented as ΔEMM with 95% confidence intervals (95% CIs), n is the number of measurement pairs. A negative ΔEMM denotes a decrease over time.

		Model 2
T	NO_X quartile	ΔEMM (95% CI)
1	1	ref
1	2	2.15 (-2.78-7.08)
1	3	-0.35 (-4.59-3.89)
1	4	1.14 (-2.99-5.28
2	1	ref
2	2	0.96 (-1.28-3.20)
2	3	0.80 (-1.44-3.05)
2	4	1.26 (-0.87-3.40)
3	1	Ref
3	2	1.28 (-1.40-3.97)
3	3	-1.33 (-3.91-1.25)
3	4	1.03 (-1.51-3.56)

Model 2 includes variables for NO_x, age, test occasion, number of total tests, and a cross-product between NO_x and test occasion, education, sex, smoking, BMI, physical activity, living with someone, and work status.
